# Intention for internal whistleblowing to report sexual violence in higher education institutions: a Nigerian national study

**DOI:** 10.12688/f1000research.141545.3

**Published:** 2024-06-27

**Authors:** Adesola A. Ogunfowokan, Saleh N. Garba, Monica A. Orisadare, Ayobami G. Adeleke, Patience E. Samson-Akpan, Mathew O. Olatubi, Omowumi R. Salau, Ayotunde Titilayo

**Affiliations:** 1 Department of Nursing Science, Obafemi Awolowo University, Ile-Ife, Osun State, Nigeria; 2 Department of Nursing Science, Bayero University, Kano, Nigeria; 3 Department of Economics, Obafemi Awolowo University, Ile-Ife, Osun State, 220005, Nigeria; 4 Institute of Education, Obafemi Awolowo University, Ile-Ife, Osun State, 220005, Nigeria; 5 Department of Nursing Science, University of Calabar, Calabar, Cross River State, Nigeria; 6 Faculty of Nursing Science, Bowen University, Iwo, Osun State, Nigeria; 7 Clinical Nursing Unit, Royal Bournemouth Hospital, Bournemouth, England, UK; 8 Department of Demography and Social Statistics, Obafemi Awolowo University, Ile-Ife, Osun State, 220005, Nigeria

**Keywords:** Higher education; Sexual violence; Rape, Sexual Harassment, Attempted rape, Dataset, Whistleblowing.

## Abstract

**Background:**

Sexual violence is prevalent in higher education institutions in Nigeria and stakeholders have encouraged staff and students to blow the whistle whenever they fall victim to or are aware of any sexual violence case. However, there is lack of data about whether the staff and students of these institutions have the intention to blow the whistle internally (within the institution) or not. There is also a lack of data on the existing reporting mechanisms or preferred whistleblowing mechanisms in these institutions. These have hindered the analysis of stakeholders’ opinions on this topic.

**Methods:**

This data note presents a comprehensive quantitative and qualitative data set collected from staff and students of three categories of government owned higher education institutions (Universities, Polytechnics, Colleges of Education) in Nigeria. Data collection was between February and December, 2021, during which quantitative data were collected from 21,937 students and 3,108 staff. Qualitatively, 138 students and 111 staff participated in a total of 35 focus group discussion sessions. The study provides unique information on respondents’ attitude, self-efficacy, and subjective norm to sexual violence whistleblowing. It also provides information on self-reported sexual violence experiences, whistleblowing intention, reporting systems in higher institutions and the preferred sexual violence whistleblowing mechanisms.

**Conclusions:**

In this data note, we provide a detailed account of the variables in the dataset and then highlight the potential of this study to contribute to improved sexual violence reporting in higher education institutions, thereby reducing the occurrence of the social menace.

## Introduction

Sexual violence has been a global issue for many years, most especially in the 21 ^st^ century among the young population. Students of higher education have been reported to fall victim to and have also been documented to be perpetrators of this social menace. In Nigeria, there are reports of increased prevalence of sexual violence among higher education students ( Egbegi *et al.*, 2019; Yesufu & Adimula, 2018, April, 23; Onoyase, 2019; British Broadcasting Corporation - BBC, 2019). Due to the frequency of sexual violence in educational institutions in Nigeria, the Independent Corrupt Practices and other Related Offences Commission (ICPC) (an agency established to prosecute perpetrators of corrupt practices in Nigeria) instructed students to report cases of sexual harassment to the commission ( ICPC, 2019) and that such cases will be handled under anti-corruption laws. Anecdotal evidences have shown that, the reporting systems for sexual violating acts have not been very effective in many of these institutions as many cases still go unreported, thereby perpetuating the menace. As far back as 2004, Lee and others have documented that whistleblowing can be used to report sexual harassment just like any other organizational wrongdoings ( Lee *et al.*, 2004). There has been a call for the use of internal whistleblowing for reporting sexual violence in higher education institutions in Nigeria by many stakeholders. Whistleblowing is an act of disclosing information that are considered to be of public interest and are also unethical ( Dungan, 2015). The act of whistleblowing is generally backed by law which also gives protection to the whistleblower ( Latan, 2023). This is quite different from reporting in which issues are brought to the attention of authorities and such issues may not have serious public concern or legal backing ( King III, 2000). Most times in Nigerian educational institutions, students and staff are regularly encouraged to report acts of sexual violence but, there is a shift now from reporting to whistleblowing because, it’s been observed that cases of sexual violence is still rampant in educational institutions. Less than two years ago, the Shehu Musa Yar’Adua Foundation, in partnership with the Gender and Development Policy Centre, University of Nigeria Nsukka, launched the ’Gender Justice Whistleblowing Portal’ for reporting sexual violence at the university ( Odu, 2021; Nov. 19). 

As a result of the level of seriousness attached to whistleblowing, we are of the opinion that an individual must have intention to blow the whistle for sexual violence before carrying out the task. An enabling environment or recognized laid down mechanism must be put in place to ensure effective whistleblowing. As of today, we are not aware of any dataset that provides information on all of these variables which may subsequently be analysed and used for policy formulation for sexual violence prevention in higher educational institutions in Nigeria. Therefore, this data note provides information on the variables included in the study dataset which are: respondents’ attitude, self-efficacy, subjective norm; self-reported sexual violence experiences; whistleblowing intention, reporting systems in higher institutions and the preferred sexual violence whistleblowing mechanisms. In this data note, we provide detailed account of the variables in the dataset and then highlight the potential of this study to contribute to improved sexual violence reporting in higher education institutions thereby reducing the occurrence of the menace.

### Specific objectives of the study

The six specific objectives of the study were to

1. describe the sexual violence experiences of students in tertiary educational institutions;
2. determine the influence of attitude, subjective norm and perceived behavioural control on intentions of students and staff to internally blow the whistle for reporting sexual violence cases;
3. identify socio-demographic factors (academic level, course of study, gender, age, sexual violence experiences, tribe and religion) that predict students’ intention to internally blow the whistle for reporting sexual violence cases;
4. identify socio-demographic factors (gender, age, tribe, religion, designation and length of service) that predict staff intention to internally blow the whistle for reporting sexual violence cases;
5. explore possible internal whistleblowing strategies for reporting sexual violence cases; and
6. explore strategies for protecting sexual violence whistleblowers.

### Theoretical framework

**Figure 1.**
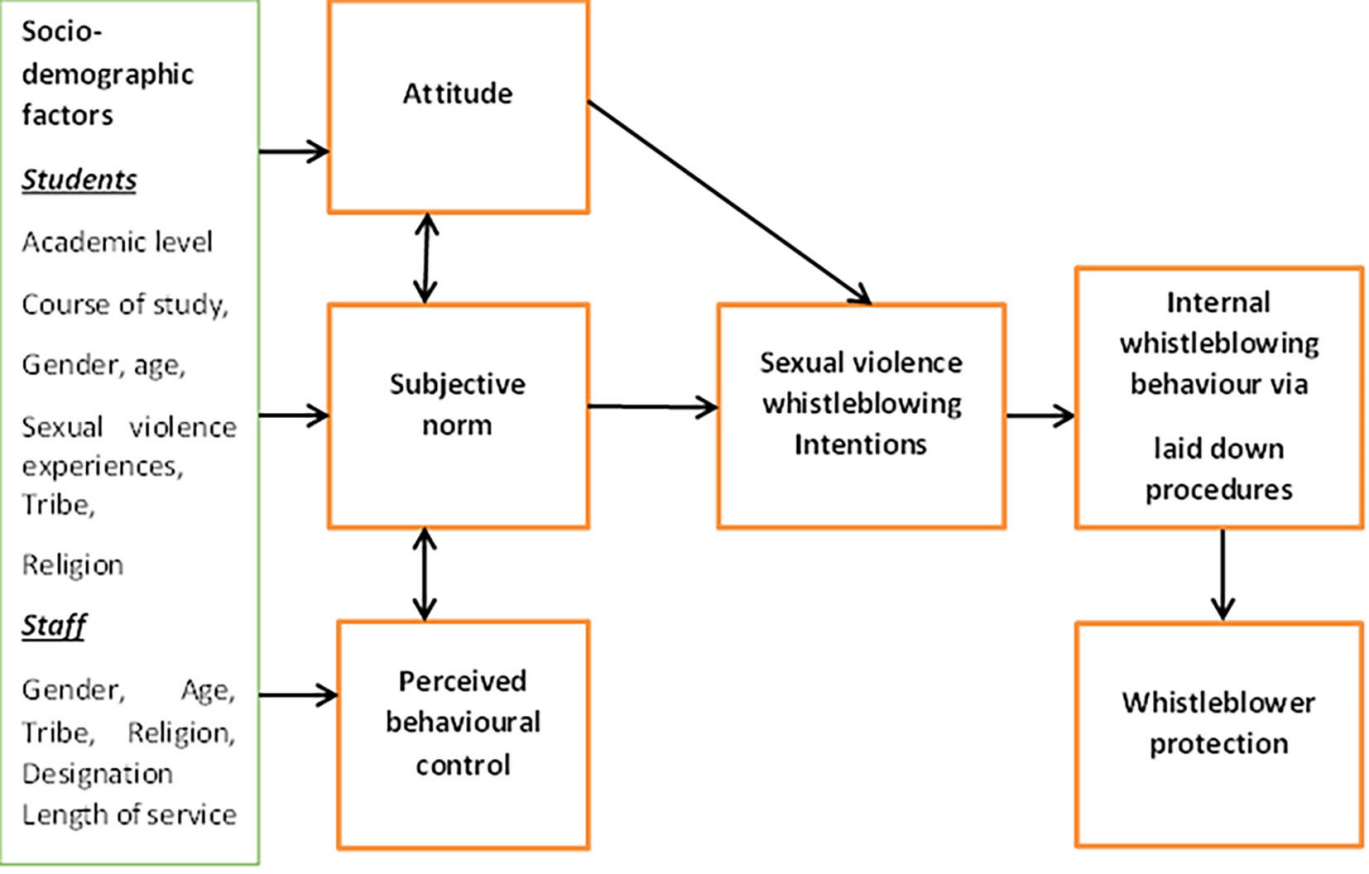
Framework for the study.

This study was hinged on the Theory of Planned Behaviour (TPB). The theory describes that behavioural intention of an individual to engage in a particular behaviour is a product of three determinants: the individual’s attitude (opinions of oneself towards the behaviour); the subjective norms (opinions of others towards the behaviour); and perceived behavioural control (self-efficacy towards the behaviour) ( Ajzen, 2012). According to Arafat and Ibrahim’s (2018), the greater the subjective norm, the favorable behavior (attitude), and perceived control, the stronger the person’s intention to perform the behavior in question. Whistleblowing has been referred to as a planned behaviour, and the Theory of Planned Behaviour has been used by several authors, such as Tarjo *et al.* (2019), Zakaria *et al.* (2016), Owusu *et al.* (2020), and Wahyuni *et al.* (2021), to support whistleblowing intention. In the Nigerian context, for an individual to have intention for whistle blowing for reporting sexual violence act, he/she must have a positive attitude towards whistleblowing as an effective mechanism. Such individual must also consider that, his/her action will be acceptable by significant others and that he/she has the capacity and the wherewithal to blow the whistle. This is so because, reporting perpetrators of sexual violence in Nigeria is considered an unsafe action for the reporter or the survivor ( Aborisade, 2014). Aside from the three determinants of TPB, background variables such as demographic factors have been identified to indirectly influence behavioural intention through the three determinants and consequently the behaviour ( Menozzi *et al.,* 2015). It is expected in this study that the socio-demographic factors of the students and staff such as academic level, course of study, gender, age, sexual violence experiences, tribe, religion, designation and length of service will influence their intention to internally blow the whistle for sexual violence ( Figure 1).

## Methods

A concurrent mixed method design using quantitative and qualitative data collection methods was adopted for the study. The study was carried out in 18 institutions comprising Federal Colleges of Education, Polytechnics and Universities in all the six geo-political zones of Nigeria. The target population of the students in the selected schools was 246,216 students while that of staff was 31,982 ( National University Commission - NUC, 2017; Federal Ministry of Education, 2019; National Bureau of Statistics, 2019). The sample size calculation was in two modes; qualitative and quantitative methods.

### Eligibility criteria

Institutions where the study was conducted were those owned and controlled by the Federal Government of Nigeria and must either be a polytechnic, college of education or university. The students were those undergoing full-time traditional face-to-face programmes and were undergraduates. Staff participants were those who work full-time mode in the selected institutions. Contract or part-time staff were excluded from the study.

### Quantitative strand

Using Cochran’s survey sample size formula: n = z ^2^pq/e ^2^ where n = sample size; z = standard normal deviate set at 1.95 which corresponds to 95% confidence level; p = 0.5 (set at 50% for unknown prevalence of whistleblowing intention); q = 1-p = 0.5; e = permitted error of margin set at 2.5% (in order to get a large representative sample), a sample size of 1,521 students was obtained per institution. Adding non-response rate of 10%, a final sample size of 1,673 was obtained per institution giving a total of 30,114 students’ respondents for the 18 institutions. Using the same Cochran’s formula: n = z ^2^pq/e ^2^ and same parameters for the staff except for the permitted error of margin set at 5%, a sample size of 418 was obtained per institution after adding 10% non-response rate. Therefore, a total of 7,524 staff (academic & non-academic) was selected and distributed proportionately in all the 18 institutions and into equal proportion of male and female staff.

### Qualitative strand

The number of participants in non-commercial focus groups is recommended to be between six and 10 in order to be able to control the group ( Kabir, 2016). Therefore, we proposed to have eight members (four males and four females) of the Student Union Government in each of the institutions to participate in the study making a total of 144 students for the 18 institutions. Also, two representatives (a male and a female) from each of the Staff Unions in each institution were selected to participate in the FGD. The Unions represented are listed according to the type of school:

• University: Academic Staff Union of Universities (ASUU); Senior Staff Association of Nigerian Universities (SSANU); National Association of Academic Technologies (NAAT); and the Non-Academic Staff Union of Universities (NASUU).
• Colleges of Education: Colleges of Education Academic Staff Union (COEASU); Senior Staff Union of Colleges of Education in Nigeria (SSUCOEN); and Non-Academic Staff Union of Colleges of Education (NASUCED).
• Polytechnics: Academic Staff Union of Polytechnics (ASUP); Senior Staff Association of Nigerian Polytechnics (SSANIP); and Non-Academic Staff Union of Polytechnics (NASUP).

Therefore, for Universities, there were eight executive officers (four males, four females) from each institution giving a total of 48 participants for the six Universities. For Colleges of Education and Polytechnics, there were six officers (three males and three females) from each institution giving a total of 72 participants for the 12 institutions. Overall, a total of 120 staff formed the sample size for the staff FGD participants.

### Sampling

**Figure 2.**
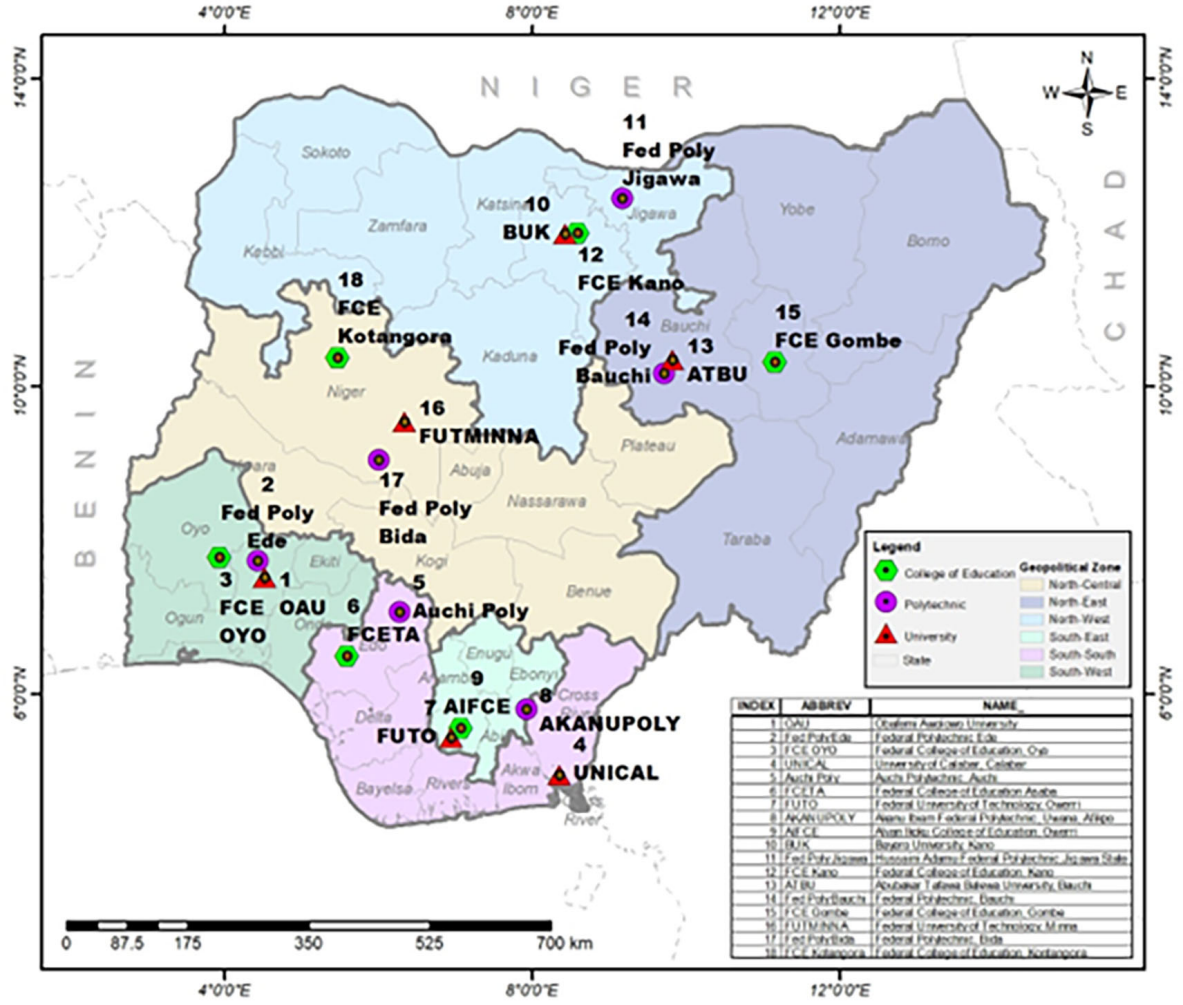
Map of the six geopolitical zones showing selected schools for the study. Source: the authors.

Multistage stratified sampling technique was adopted in this study. Nigeria is already stratified into six geo-political zones and in each zone, government-owned higher institutions were stratified by University, Polytechnic and College of Education. In each of the stratum, one institution was selected using simple random sampling technique (balloting) thereby giving a total of three schools from each zone ( Figure 2 - produced by the authors). A random selection of departments from each of the schools were done followed by the students’ and staff stratification into male and female gender and also stratification into academic and non-academic staff. A random selection was finally used to select the respondents by proportion as shown in Table 1. For the qualitative strand, the Presidents of each of the unions were contacted to nominate members of their cabinet to participate in the FGD putting into consideration equal gender in the nomination. However, it was observed that there were more males in many of the cabinets than females hence, more male officers participated in the FGD than females. This has been attributed to the general assumption that more males take on leadership roles than females.

**Table 1.** Distribution of sample size of students and staff by institutions.

Region	State	School	Student population	Sample	Staff population	Sample
South-West	Oyo	Federal College of Education, Oyo	6,075	743	1,021	240
	Osun	Federal Polytechnic, Ede	7,655	936	800	188
	Osun	Obafemi Awolowo University, Ile-Ife	29,938	3,662	1,723	405
South-South	Asaba	Federal College of Education, Asaba	5, 047	617	226	53
		Auchi Polytechnic	8,376	1,025	1,324	312
	Cross-River	University of Calabar, Calabar	29,566	3,616	6,278	1,477
South-East	Imo	Alvan Ikoku College of Education, Owerri	7,909	967	1,554	366
	Ebonyi	Akanu Ibiam Federal Polytechnic, Uwana, Afikpo	4,244	519	251	59
	Imo	Federal University of Technology, Owerri	20,924	2,559	3,107	731
North-West	Kano	Federal College of Education, Kano	8,200	1,003	2,284	537
	Jigawa	Hussaini Adamu Federal Polytechnic Kazaure	4,478	548	132	31
	Kano	Bayero University, Kano	34,912	4,270	4,289	1,009
North-Central	Niger	Federal College of Education, Kotangora	3, 928	481	611	144
	Niger	Federal Polytechnic, Bida	5,819	712	229	54
	Niger	Federal University of Technology, Minna	18,407	2,251	3,481	819
North-East	Gombe	Federal College of Education, Gombe	24,773	3,030	768	181
	Bauchi	Federal Polytechnic, Bauchi	9,773	1,195	1,477	348
	Bauchi	Abubakar Tafawa Balewa University, Bauchi	16,191	1,980	2,427	571
**Total**			**246,216**	**30,114**	**31,982**	**7,524**

### Instruments

Two instruments were used for data collection: Sexual Violence Whistleblowing Intentions Questionnaire (SeVWIQ) and a Focus Group Discussion Guide.

1. The SeVWIQ was in two forms. The first was administered to students and it elicited information on their attitude to whistleblowing, subjective norm, self-efficacy, whistleblowing intentions, sexual violence experiences and demographics. The second was for the staff and it elicited information on their attitude to whistleblowing, subjective norm, self-efficacy, whistleblowing intentions, and demographics. The questionnaire had 10 attitudinal questions on a 3-point Likert scale of Not True (1), Moderately True (2) and Very True (3). The subjective norm questions were nine on a 3-point Likert scale of Not Proud (1), Slightly Proud (2) and Very Proud (3). The self-efficacy questions were six on 3 point-Likert scale of Not Likely (1), Moderately Likely (2) and Very Likely (3). The questions on the attitude, subjective norm and self-efficacy were adapted from the work of Zakaria *et al.* (2016). Three questions were constructed to represent acts of rape, attempted rape and sexual harassment which were used to assess the sexual violence experiences of the students. The students were asked to state the number of times (0, 1, 2, 3+) they experienced the acts in the past 12 months. The essence of this sexual violence experience section of the questionnaire was to confirm in our study that sexual violence still occurs among higher education students. We did not go to the field to conduct full sexual violence prevalence study since there are many studies that have done this in Nigeria. The whistleblowing intention component of the questionnaire were formulated using three vignettes which were adapted from the work of Ahmad (2011). The vignettes represented cases of rape, attempted rape and sexual harassment and were rated on a scale of 1 to 5. Information on the vignettes depicted the participants as third-party whistleblowers and not as survivors. It is assumed that survivors will report when they are victimised, but a third-party report is not the usual practice in higher education institutions (HEIs). Whistleblowing action can be taken by a third-party and not necessarily the survivor, and we are of the opinion that the HEIs should be considering third-party whistleblowing and also take action when there is evidence.
2. The Focus Group Discussion Guide (FGDG) which was developed from the review of literature, explored information on sexual violence cases on the school campuses, institutional laid down strategies for reporting sexual violence, factors that may predict the staff and students’ intention to blow the whistle, strategies for reporting and protecting whistleblowers, and the effectiveness of internal whistleblowing strategies.

### Pilot study/pre-test of instruments

The pilot study was carried out in a University of Technology in South West Nigeria. Using the recommendation of Connelly, (2008) that 10% of main sample size can be used for pilot study, therefore, 167 students (10% of 1,673) and 42 staff (10% of 418) were selected to participate in the pilot study. Eight members of the Student Union Government and eight members of the leadership of the four Staff Unions of the school participated in the two separate FGD sessions (one for the students and the other for staff). During the pilot study, the officers of the Academic Staff Union of Universities (ASUU) in the school did not participate in the FGD despite various efforts to convince them to participate. We therefore recruited academic staff members who were not necessarily officers of the union to participate in the study. During the main study, we planned to use this method in any of the schools peradventure we ran into similar problems. Fortunately, we never experienced such problem except in a particular school where the entire officers of the unions, both academic and non-academic, refused to participate hence, we did not conduct FGD in such school. During the pilot study, the questionnaire was subjected to face and content validity and internal consistency, while trustworthiness of the FGD data was also ensured. Part of the findings from the pilot study has been published in Ogunfowokan *et al.* (2023). Also, the questionnaire’s internal consistency was further assessed during the main study to further evaluate its reliability. The Cronbach alpha results for both the pilot and the main studies are stated in Table 2.

**Table 2.** Cronbach’s alpha statistics for study questionnaire during the pilot and main studies.

Variables	Students		Staff	
	Pilot	Main study	Pilot	Main study
Attitude	0.797	0.807	0.770	0.805
Subjective norm	0.628	0.817	0.819	0.831
Self-efficacy	0.804	0.776	0.629	0.796
Whistleblowing intention	0.734	0.857	0.663	0.841
Sexual violence experiences	0.478	0.795	**--**	**--**

### Field staff

A field supervisor was identified in each of the schools except the three Northern regions where only one field supervisor was identified and he worked with the investigator from the North to collect the data. The supervisors were responsible for the logistics of sampling and the data collection process. They were trained twice using the virtual mode between December 2020 and January, 2021. The principal investigator was the chief trainer supported by other investigators. The field supervisors then appointed field workers in their respective schools and were trained in the administration of the questionnaires. Also, the two mentees who were postgraduate students as required in the funding call served as the research assistants for the study. The mentees have been part of the writing process for the grant proposal and they were further trained alongside the supervisors.

### Ethical considerations

Institutional board approval for the study was obtained on 11 ^th^ December, 2019 from the Institute of Public Health, Health Research and Ethics Committee (IPH HREC) of the Obafemi Awolowo University with approval number IPH/OAU/12/1460. An extension of the approval was obtained on 5 ^th^ May, 2021 at the expiration of the initial approval when data collection has not been completed. Informed consent of the students and staff was obtained using the informed consent form formatted after the IPH HREC form. The participants were briefed on the study as a whistleblowing intention study. They were assured of their privacy and the confidentiality of their information. The informed consent forms also contained information on how their privacy and confidentiality would be assured. They were given the option of withdrawing from the study if they felt very uncomfortable during the process of data collection. The participants’ informed consent was obtained by the field staff after the purpose of the study was explained and concerns clarified. However, due to the sensitivity of the questions, many participants declined appending their signatures on the consent form but preferred verbal consent without recording, which was granted them. In addition, written permission to collect data was also obtained from the administrators of the respective study institutions.

#### Addressing the emotional impact of the study

During the planning phase of data collection, the team had a relationship with each of the university health centres via the institutions’ administrators. There was a plan to refer any student or staff who may be emotionally unstable as a result of their past sexual violence experience to the school health centre, where they would be managed appropriately. Luckily, we didn’t record any of such events; however, we received a particular questionnaire where a student wrote on it, “Ma, thank you for conducting this study; I had an experience of this with my father about two years ago.” Unfortunately, we could not trace this participant because the questionnaire was de-identified. Also, our interaction with the field supervisors during their training showed that they were excited about the study, and none of them expressed any emotional disturbance resulting from their past victimisation experience. During the training of the field supervisors, efforts were made to inform them of the likely emotional reactions of some participants who must have been survivors of sexual violence. They were encouraged to refer such participants to the university health centre, where they would be managed appropriately. The field supervisors in turn trained the field workers that worked under them.

### Data collection process

Due to the sensitivity of the study and the non-appealing nature of electronic data collection to many Nigerians as previously observed ( Tella, 2015), we decided to use the paper-based questionnaire administration methods. The selected students were contacted by the field workers in their various lecture rooms and questionnaires were administered to them and same were retrieved immediately after completion. Some of them requested to return the questionnaire the following day. Also, some class representatives assisted in retrieving the questionnaires from those who made such request. The class representatives collecting filled-out questionnaires from participants was not a study option but an individual option. Some of the students insisted they could not fill and submit the questionnaires immediately; hence, they requested that they would submit to their class representatives for onward submission to the field workers. All efforts to dissuade them from this proved abortive, and we needed to respect their decisions. However, our observation on the field is that some of the participants did not see their responses as so confidential that their colleagues could not have access to them, more so that the bulk of the questions were on whistleblowing intention.

The questionnaires for the staff were administered to them in their various offices. Some of them returned the questionnaires immediately after completion while others requested to return them after some days. Some staff also dropped the questionnaires in their pigeon holes for pick up. In addition, two FGD sessions (one for students and the other for staff) were conducted in each of the schools giving a total of 35 FGD sessions (the staff in a particular school refused to participate in the FGD). For the students’ FGD, they were carried out at the Student Union Government building except for one of the schools in which the school management insisted it should be conducted at the Students’ Affairs building. The staff FGD were conducted at a designated venue agreed-on between the field supervisors and the participants. The principal investigator facilitated the sessions in support of other team members for the Southern regions. For the Northern regions, the field supervisor and the investigator from the North facilitated the FGD sessions. Recordings of the sessions were taken using Android phones while field notes were taken by other support staff. While the discussion was ongoing, the facilitator reflected on the statements of the participants to confirm their true position. After the discussion, members were asked to exercise some patience while they listened to the selected part of the interview as deemed fit by the facilitator. In fact, in a particular school, the staff group agreed and insisted that a participant must be allowed to record the discussion before the discussion can commence. According to them, this act is to ensure that they had evidence of what the discussion was all about if peradventure, anything goes wrong in the future.

The mixed-gender focus group discussion was adopted because, in class settings in higher education institutions, sexual violence is usually discussed among the students irrespective of their gender. We did not record any issue with the mixed-gender group. The heterogenity 4 of the group is based on participants being officers of students’ and staff’s associations. It is a general observation that males take on more leadership roles than females, which explains why there were more males in the group than females.

### Data entry

Out of the 30,114 students’ questionnaires and 7,524 staff questionnaires administered, a total of 21,937 students’ questionnaires and 3,108 staff questionnaires were completely and properly filled and were adequate for data analysis giving a response rate of 72.8% for students and 41.3% for staff. There were eight data entry clerks who were recruited and trained in June, 2021. Two data analysts were also employed for the quantitative strand and these analysts trained the data entry clerks on how to enter the coded data from the questionnaires into Epi Info software version 3.1. Hands-on training was done using pre-coded demo-questionnaires. All the questionnaires were numbered and coded for data entry. The clerks received different quantities of questionnaires on different days for entry into Epi info software. A standby research office assistant was also employed to assign the questionnaires to the clerks and also ensured that correct quantities of the questionnaires were returned after data entry. Data entry was done within a period of seven months. Following the entry of the data and the merging of the data files from the clerks by the analysts, the data were then exported to SPSS version 27 ( IBM Corp. Released, 2020).

### Dataset

The quantitative dataset ( Ogunfowokan *et al*., 2023) consists of 44 variables for the students and 42 variables for the staff that are grouped into six: (i) attitude to whistleblowing; (ii) subjective norm; (iii) self-efficacy; (iv) whistleblowing intention; (v) sexual violence experience; and (vi) socio-demographic characteristics. However, the staff dataset does not contain the sexual violence experience data. The list of all the variables and their corresponding response options are found in Tables 3 and 4. The qualitative dataset contains transcripts on responses to the six probing questions used for the FGD which are: (i) sexual violence cases in the school; (ii) institutional reporting systems; (iii) factors influencing intention; (iv) preferred whistleblowing strategies; vi) protection of whistleblower; and (vii) effectiveness of internal whistleblowing.

**Table 3.** Variables included in the students’ dataset.

Variable	Question number
**Attitude to whistleblowing**	
Whistleblowing reduces incidence of sexual violence	A1
Reporting sexual violence controls perpetration	A2
Whistleblowing enhances the public interest	A3
Whistleblowing for sexual violence reporting is to prove loyalty to the school	A4
Whistleblowing is the moral thing to do	A5
Whistleblowing enhances organization’s sustainability	A6
Whistleblowing is a way of exercising true conscience	A7
Whistleblowing is to enable student to be a moral agent	A8
Whistleblowing is the management tool to protect students	A9
Whistleblowing is to make perpetrators liable for their wrong doings	A10
**Subjective norm**	
Classmates	B11
Course-mates	B12
Family members	B13
Immediate Supervisor	B14
Lecturers	B15
Neighbours	B16
The public	B17
Friends	B18
School Administrators	B19
**Self-efficacy**	
School will ignore my reporting	C20
My reporting won’t make any difference	C21
I will be subjected to harassment by my colleagues	C22
I will be isolated by my friends	C23
1 will be closely monitored	C24
I will be charged for breach of loyalty to the school	C25
**Whistleblowing intention**	
Rate the extent to which the behaviour is offensive	D26
Rate the likelihood that you will report	D27
Rate the likelihood that your schoolmates/classmates will report	D28
Rate the extent to which the behavior of the course-mate is offensive	D29
Rate the likelihood that you will report	D30
Rate the likelihood that your schoolmates/classmates will report	D31
Rate the extent to which the behaviour is offensive	D32
Rate the likelihood that you will report	D33
Rate the likelihood that your schoolmates/classmates will report	D34
**Sexual violence experiences**	
Number of time attempted to be rape in the past 12 months	E35
Number of time raped in the past 12 months	E36
Number of time sexually harassed in the past 12 months	E37
**Socio-demographic variables**	
Gender	F38
Religion	F39
Age	F40
Trobe	F41
Course of Study	F42
Faculty/Field	F43
Level	F44
Name of Institution	
Type of Institution	
Region	

**Table 4.** Variables included in the staff dataset.

Variable	Question number
**Attitude to whistleblowing**	
Whistleblowing reduces incidence of sexual violence	A1
Reporting sexual violence controls perpetration	A2
Whistleblowing enhances the school community interest	A3
Whistleblowing for sexual violence reporting is to prove loyalty to the school	A4
Whistleblowing is the moral thing to do	A5
Whistleblowing enhances organization’s sustainability	A6
Whistleblowing is a way of exercising true conscience	A7
Whistleblowing is to enable employee to be a moral agent	A8
Whistleblowing is the management tool to protect students	A9
Whistleblowing is to make perpetrators liable for their wrongdoings	A10
**Subjective norm**	
Co-workers	B11
Superior	B12
School management	B13
Subordinate	B14
Office mate	B15
Neighbours	B16
The public	B17
Friends	B18
Family members	B19
**Self-efficacy**	
School will ignore my reporting	C20
My reporting won’t make any difference	C21
I will be subjected to harassment by my colleagues	C22
I will be isolated by my friends	C23
I will be closely monitored	C24
I will be charged for breach of loyalty to the school	C25
I will be demoted	C26
**Whistleblowing intention**	
Rate the extent to which the behaviour is offensive	D26
Rate the likelihood that you will report	D28
Rate the likelihood that your colleagues will report	D29
Rate the extent to which the behavior of the course-mate is offensive	D30
Rate the likelihood that you will report	D31
Rate the likelihood that your colleagues will report	D32
Rate the extent to which the behaviour is offensive	D33
Rate the likelihood that you will report	D34
Rate the likelihood that your colleagues will report	D35
**Socio-demographic variables**	
Gender	E36
Religion	F37
Age	F38
Trobe	F41
Course of Study	F42
Faculty/Field	F43
Level	F44
Name of Institution	
Type of Institution	
Region	

### Dataset validation

During sampling of the schools, some areas in the Northeast and Northwest regions were avoided because of insurgency. After sampling, two schools were dropped because the field workers could not gain access to the schools due to ongoing insurgency in the areas. Also the study was conducted in government owned schools thereby excluding higher institutions owned by private individuals, organizations and State Governments. Other higher institutions like diploma schools of nursing, technical and specialized higher education schools among others were also not captured in the study. Hence, generalization of the findings from this study should only be for Universities, Polytechnics and Colleges of Education that are Federal government-owned. Also, FGD for staff could not be conducted in a particular university because the executive officers for the staff unions did not give their consent to participate despite all pleas and provision of information about the study.

*Quantitative dataset*

The questionnaires that were brought from the field were assessed for completeness by the research office assistants and two other young people employed for the job. All incompletely filled questionnaires were discarded. Consistency checks were done by ensuring that the data on each of questionnaires were consistent. For example, on the staff questionnaire, an academic staff who reported to be a Senior Lecturer later ticked the non-academic status had the non-academic status corrected. Duplicate and outliers on the dataset were also observed and compared with the original raw data on the corresponding questionnaires and corrections were made where necessary. Missing data were attended to by using mean substitution as documented by Kang (2013). However, we were aware of the bias related to self-reported data collection most especially on a sensitive issue as sexual violence and this may impact on the quality of the dataset. Also, we could not do external validation of the data set as we could not lay our hands on a similar dataset on any of the variables.

*Qualitative dataset*

A most profound validation for the qualitative dataset that we carried out is the data member checking. During and after the interview, participants were regularly instructed to confirm their statements as true reflections of their opinions by listening to the recordings. Facilitators were also encouraged to clarify participants’ opinions from the statements that they made to avoid biases. Peer debriding was also done among the investigators, supervisor and the research assistants immediately after concluding an FDG session to discuss the findings in proper perspective and to also avoid biases. All these efforts were to ensure the trustworthiness of the qualitative data ( Stahl and King, 2020).

### Reflexivity statement

There are six investigators and two research mentees in the research team. The team consists of individuals who are academics from four universities spread across three regions in Nigeria. The research mentees were Ph.D. students (a male and female) in community health nursing, of whom one of them focused on adolescent boys’ sexual violence perpetration in her Ph.D. project while the other focused on work-related stress among nurses. Both mentees were trained in the use of qualitative and quantitative data collection methods for their M.Sc. and Ph.D. projects. The six investigators (three males and three females) had Ph.D. certificates in diverse specialised areas, which include health promotion, civic education, gender and policy studies, demography and population studies, medical sociology, and community health. Some of the investigators were trained in phenomenology studies, while others were trained in quantitative data collection. The strengths of the investigators were identified and made use of during data collection. The principal investigator, who is a community health nurse expert, facilitated the FGD sessions in the southern regions. The assigned field supervisor for the Northern region, who is a sociologist and a demographer, facilitated the FGD for the Northern region in conjunction with the investigator from the North, who is a medial sociologist. All these investigators had at one point in time involved in quantitative and qualitative studies.

### Limitations

Some schools in the northern part of the country were intentionally avoided as a result of the insurgency going on in those areas at the time of conducting the study. Also, two schools that were sampled initially were replaced at the point of data collection when the field workers could not gain access to the schools due to insurgency. Other higher institutions, like diploma schools of nursing technical schools and specialised higher education schools, among others, were not captured in the study. Hence, generalisation of the findings from this study should only be made for students in the universities polytechnics, and colleges of education. The staff in one of the institutions refused to participate in the FGD despite all efforts to convince them that the study is solely for research purposes with no implication for any individual.

## Data Availability

*Qualitative data* The underlying qualitative data (transcripts) are not made publicly available due to the sensitivity of the information. Any request for the data should be made to the corresponding author at solafowokan@oauife.edu.ng or adesolaogunfowokan@gmail.com. The applicant for the dataset must be affiliated with a research or academic institution and must explicitly indicate the purpose of the dataset request. The qualitative dataset will be made available following de-identification of the set. *Quantitative data* The dataset can be found at Zenodo repository via this link - https://zenodo.org/records/10019951. The link also contains the blank questionnaires and focus group discussion guide used in the study. Data are available under the terms of the Creative Commons Attribution 4.0 International license (CC-BY 4.0).
