# Supplementary material for: Intention for internal whistleblowing to report sexual violence in higher education institutions: a Nigerian national study
Source: F1000Res. 2024 Jun 27;12:1351. Originally published 2023 Oct 17. [Version 3] doi: 10.12688/f1000research.141545.3 (PMC11350322; doi:10.12688/f1000research.141545.3)
Supplement: Peer review history

# Reviewer report: Reviewer response for version 3

**DOI:** 10.5256/f1000research.168217.r296254

**Published:** 2024-07-10

**Authors:** Anna Bull

**Affiliations:** 1 Department of Education, University of York, York, England, UK

Thank you for these revisions.

Are sufficient details of methods and materials provided to allow replication by others?

Yes

Is the rationale for creating the dataset(s) clearly described?

Partly

Are the datasets clearly presented in a useable and accessible format?

Yes

Are the protocols appropriate and is the work technically sound?

No

Reviewer Expertise:

Gender-based violence in higher education; sexual harassment; gender inequalities; qualitative and quantitative research

I confirm that I have read this submission and believe that I have an appropriate level of expertise to confirm that it is of an acceptable scientific standard.

# Reviewer report: Reviewer response for version 2

**DOI:** 10.5256/f1000research.165930.r274864

**Published:** 2024-06-12

**Authors:** Anna Bull

**Affiliations:** 1 Department of Education, University of York, York, England, UK

Thank you for carrying out extensive revisions to improve the transparency of this study. The further detail on ethics, theoretical framework, and methods are very helpful. However, there is still one point that needs to be amended before I can approve this study. 

 The section on the survey instrument states that:

 'The sexual violence experiences component was adapted from the Sexual Experiences Short Form Victimization (SES-SFV) scale developed by Koss *et al.* (2006).'

 However, the questions in the survey instrument read as follows:

 35. In the past 12 months, how many times

 has someone attempted to rape you?

 0, 1, 2, 3+

 36. In the past 12 months, how many times has someone actually raped you?

 0, 1, 2, 3+

 These questions are not compatible with the  Sexual Experiences Short Form Victimization (SES-SFV) scale, as they use the term 'rape', which the SES-SFV survey does not. Therefore, please remove any reference to use of the Sexual Experiences Short Form Victimization (SES-SFV) scale from the study, as this is inaccurate.

 For clarity of terminology, please also briefly add in an explanation of the difference between 'whistleblowing' and 'reporting' as noted in my previous review. If these terms have the same meaning in your study, please explain this.

Are sufficient details of methods and materials provided to allow replication by others?

Yes

Is the rationale for creating the dataset(s) clearly described?

Partly

Are the datasets clearly presented in a useable and accessible format?

Yes

Are the protocols appropriate and is the work technically sound?

No

Reviewer Expertise:

Gender-based violence in higher education; sexual harassment; gender inequalities; qualitative and quantitative research

I confirm that I have read this submission and believe that I have an appropriate level of expertise to confirm that it is of an acceptable scientific standard, however I have significant reservations, as outlined above.

# Response

**Published:** 2024-06-20

**Authors:** Adesola Ogunfowokan

**Affiliations:** Nursing Science, Obafemi Awolowo University, Ife, Osun, Nigeria

Thank you for creatng time to review this second version of the article. In accordnace to your comments, the following revisions were made.

 - The statement indicating the adaptation of the SES-SFV scale in the study has been reworded

 - The difference between whistleblowing and reporting has been added to the introduction section

 - Appropriate references have also been place in the reference list

# Reviewer report: Reviewer response for version 2

**DOI:** 10.5256/f1000research.165930.r274866

**Published:** 2024-05-15

**Authors:** Florence Okoro

**Affiliations:** 1 School of Nursing, The University of North Carolina at Charlotte, Charlotte, North Carolina, USA

There no new comments from me.

Are sufficient details of methods and materials provided to allow replication by others?

Yes

Is the rationale for creating the dataset(s) clearly described?

Yes

Are the datasets clearly presented in a useable and accessible format?

Yes

Are the protocols appropriate and is the work technically sound?

Yes

Reviewer Expertise:

Behavioral science

I confirm that I have read this submission and believe that I have an appropriate level of expertise to confirm that it is of an acceptable scientific standard.

# Reviewer report: Reviewer response for version 1

**DOI:** 10.5256/f1000research.155002.r219037

**Published:** 2024-04-17

**Authors:** Florence Okoro

**Affiliations:** 1 School of Nursing, The University of North Carolina at Charlotte, Charlotte, North Carolina, USA

This is a robust study that covers all aspects of whistleblowing and sexual violence in higher institutions in Nigeria.

 The study framework - Theory of Planned Behavior aligns with the objectives of the study and the methods of data collection of the quantitative and qualitative arms of the study

 The method is sound as it covers qualitative and quantitative strands.

 Sampling is adequate - multistage stratified sampling to cover the six geopolitical zones of Nigeria.

 The data collection instruments are adequate. The SeVWIQ was administered to students and staff. The focus group discussion guide was appropriately used to facilitate the focus group discussions. The procedure for data collection was adequately described and appropriate for the study.

 The study and the report writing meet the criteria of a scientific project.

Are sufficient details of methods and materials provided to allow replication by others?

Yes

Is the rationale for creating the dataset(s) clearly described?

Yes

Are the datasets clearly presented in a useable and accessible format?

Yes

Are the protocols appropriate and is the work technically sound?

Yes

Reviewer Expertise:

Behavioral science

I confirm that I have read this submission and believe that I have an appropriate level of expertise to confirm that it is of an acceptable scientific standard.

# Reviewer report: Reviewer response for version 1

**DOI:** 10.5256/f1000research.155002.r231267

**Published:** 2024-03-04

**Authors:** Anna Bull

**Affiliations:** 1 Department of Education, University of York, York, England, UK

This data note provides a transparent overview of many aspects of the data collection and curation process for this study. It is helpful to see such a comprehensive study being carried out of sexual violence prevalence and reporting intention across a wide range of institutions. I applaud the authors for the ambition of this study as well as its scale.

 However, I have two major concerns, as well as a number of minor concerns, that mean that I can’t as yet approve it.

1. The data note states that an adapted version of the SEQ-SFV is used to explore prevalence. However, the survey design itself does not use any aspect of the SEQ-SFV and instead asks participants directly if they have been raped. This goes against good practice in sexual violence research and is likely to lead to the findings being inaccurate (see further detail below). This point needs amendment, critical discussion and explanation.
2. There is no information given relating to the ethics of carrying out research on sexual violence specifically, such as what support information was given to participants; how focus groups were held in a way to make them safe for survivors of sexual violence to participate; and what training/support was given to staff working on the project (see further detail below).

Below, I go through various points that I suggest should be addressed in this data note, including the two major issues identified above as well as various lesser concerns.

 Use of language

 In order for the article to be in dialogue with international literature in this area, there are some points of language that could be clarified. Most notably the terms ‘blow the whistle’ and ‘whistleblower’ appear to be used synonymously with reporting.

 I assume that the term ‘whistleblowing’ is the term used in Nigeria to describe reporting sexual violence to an institution. However, whistleblowing has quite a different meaning from reporting in some contexts; for example in the UK whistleblowing is a protected act, meaning that there are legal protections for whistleblowers, but speaking out only counts as whistleblowing if it is deemed to be ‘in the public interest’. Reporting individual experiences of sexual violence might not necessarily be deemed to be in the public interest. As such, further information about terminology is needed to clarify what is meant by this term.

 There are also some instances of inaccurate language or claims that could be framed more carefully:

- ‘the **magnanimity** of sexual violence in educational institutions in Nigeria’ (paragraph 1). I don’t think magnanimity is the right word here.
- ‘In cases of sexual violence, being a culturally sensitive issue, a reprisal attack on the whistleblower is inevitable.’
  - While reprisal is probably not uncommon, to say that it is inevitably is a very strong claim. I would suggest the authors qualify this claim, explain why they are taking such a strong position, or add a citation.

Theoretical framework

 The statement that ‘we are of the opinion that an individual must have intention to blow the whistle to report sexual violence before carrying out the task’ needs more explanation and needs to be positioned within wider research on reporting sexual violence. More generally, this could contribute to a justification of the use of the Theory of Planned Behavior that is used to frame the study. I’m not yet convinced that this theory is helpful in the context of this study. In fact, reporting sexual violence is sometimes opportunistic, or occurs by accident or is taken by someone other than the survivor (Lievore D, et. al. 2005 [ref 4]; Bull A, et. al. 2022 [ref 2]; Brooks-Hay et. al. 2020 [ref 1]). Furthermore, ‘intention’ in this sentence is perhaps glossing over some of the complexities of reporting; as I have outlined (Bull, 2022), there are both immediate catalysts as well as underlying rationales for reporting sexual violence or harassment to higher education institutions; this term ‘intention’ could therefore be critically discussed in more detail.

 Similarly, the claim that ‘for an individual to have intention to blow the whistle for reporting sexual violence act, he/she must have a positive attitude towards whistleblowing as an effective mechanism’ is also questionable in relation to existing literature; studies (as cited above) show that people report sexual harassment and violence to their institution primarily to protect themselves and others from further harm. Therefore it is possible that reporting parties are taking the step of reporting as they have no other options, rather than because they have a positive attitude towards reporting as an effective mechanism.

 Methods

 Qualitative methods

 The methods are clearly explained but could benefit from a rationale as to why focus group discussions were seen as appropriate, and why mixed-gender focus groups were chosen. For such a sensitive topic, as the authors note, perhaps women-only focus groups or interviews might have been more appropriate, allowing interviewees to speak more openly? This would also have overcome the issue of having more male focus group participants in the focus groups.

 In addition, the decision to hold a hypothetical discussion about whether participants would report, while a reasonable approach to take, has some limitations. The authors could therefore justify the decision to include hypothetical discussions about reporting only, and critically discuss the implications of this decision for the findings.

 Questionnaire

 The description states that the article adapted the Sexual Experiences Short Form Victimization (SES-SFV) scale. However, the questions in the questionnaire (linked at the bottom of the article) are not from the SEQ-SFV and they do not follow good practice in sexual violence research, as they use the word ‘rape’ rather than behaviorally-specific language.

 As such, it is inaccurate to state that they are an adaptation of the SES-SFV. Furthermore, the questions used will heavily underestimate prevalence of sexual violence as sexual violence survivors will often not use the term ‘rape’ even when they have experienced behaviors that do indeed fall under legal definitions of rape. This is a very serious issue in the research design which needs critical discussion and explanation.

 The Sexual Violence Whistleblowing Intentions Questionnaire needs further information; was this developed/adapted by the authors themselves? If so, could they include more detail about its development. If not, could they link to where they have obtained it from.

 The questionnaire only asks about participants’ intentions to ‘blow the whistle’ relating to third party incidents of sexual violence that they hear about. However, it is not the norm for HEIs to take action on third party reports. Therefore, it is important to explain why the authors chose to ask only about third party reporting rather than reporting by victim-survivors themselves.

 The authors state that ‘the vignettes represented cases of rape, attempted rape and sexual harassment and were rated on a scale of 1 to 5’. It is not clear what the vignettes were rated for; this becomes clear on reading the questionnaire in the linked document but it needs to be explained here.

 Ethics

 The discussion of ethical issues is very brief and omits any discussion of the ethics of sexual violence research. Questions to address include:

- What support was offered for participants who might have experienced sexual violence and found the research participation triggering?
- More reflection on the use of paper questionnaires in relation to ethics is needed. For example, if class representatives collected the questionnaires that were returned at a later point, how was confidentiality assured?
- What training did the facilitators for the focus group discussions have in carrying out research in this area (see Campbell et al., 2019 [ref 3])
- What protocols or mechanisms did the focus group discussion facilitators and organisers put in place to ensure that the discussion was safe for survivors of sexual violence? (even though the study was not directly recruiting survivors, there will of course have been some people in the sample who will have experienced sexual violence).
- Was the option to withdraw from the study available at any point, either to survey or FGD participants?
- How was the FGD schedule amended after the pilot, and were there any issues with the mixed gender groups?

Data member checking

- ‘During and after the interview, participants were regularly instructed to confirm their statements as true reflections of their opinions by listening to the recordings’
  - This point needs further explanation. Was this standard practice for all FGDs? How many members of the FGD participated in the data member checking, and what proportion of the recording did they listen back to? Were there any instances where participants’ opinions had changed since the FGD?

Reflexivity statement

- The authors state that ‘In order to eliminate any form of bias, information about the researchers was also given alongside the research information at the point of obtaining informed consent from the participants’.
  - This statement seems surprising. Eliminating any form of bias in qualitative research is not possible; rather the study design and methods should be transparently discussed so that readers can assess how the methods might influence the findings. I would recommend removing claims to avoid bias in qualitative work.

**

 I hope that these comments are helpful in enhancing the transparency and robustness of this study.

Are sufficient details of methods and materials provided to allow replication by others?

Yes

Is the rationale for creating the dataset(s) clearly described?

Partly

Are the datasets clearly presented in a useable and accessible format?

Yes

Are the protocols appropriate and is the work technically sound?

No

Reviewer Expertise:

Sexual harassment, sexual violence, higher education, gender inequalities, class inequalities, music education

I confirm that I have read this submission and believe that I have an appropriate level of expertise to state that I do not consider it to be of an acceptable scientific standard, for reasons outlined above.

## References

[1] : Doing the “Right Thing”? Understanding Why Rape Victim-Survivors Report to the Police. *Feminist Criminology* .2020;15(2) : 10.1177/1557085119859079 174-195 10.1177/1557085119859079

[2] : Catalysts and rationales for reporting staff sexual misconduct to UK higher education institutions. *Journal of Gender-Based Violence* .2022;6(1) : 10.1332/239868021X16270572218631 45-60 10.1332/239868021X16270572218631

[3] : A Trauma-Informed Approach to Sexual Violence Research Ethics and Open Science. *J Interpers Violence* .2019;34(23-24) : 10.1177/0886260519871530 4765-4793 10.1177/0886260519871530 31514606

[4] : No longer silent: A study of women's help-seeking decisions and service responses to sexual assault. *Australian Government’s Office for Women* .2005;

# Response

**Published:** 2024-03-12

**Authors:** Adesola Ogunfowokan

**Affiliations:** Nursing Science, Obafemi Awolowo University, Ife, Osun, Nigeria

Dear Dr. Bull,

 Thanks for taking the time to review this manuscript. Find below our comments on the review.

Main concerns

  

1. The study is not a sexual violence prevalence study but rather an intention study. There are many studies in Nigeria that have explored the prevalence of sexual violence among higher education students (Onayase, 2019; Duru et al., 2018; Akinbode & Ayodeji, 2018; Adogu et al., 2014; and Ado et al., 2010, to mention a few); hence, we did not see the need to conduct a full prevalence study on sexual violence using the full SES-SFV. We adapted from the scale using the response options ‘How many times in the past 12 months – 0, 1, 2, 3+. Three questions were then constructed similar to Question 10 on the scale: Have you been raped before? The three questions were constructed to represent rape, attempted rape, and sexual harassment. The essence of this was to confirm in our study that sexual violence still occurs among higher education students.

- The participants were briefed on the study as a whistleblowing intention study. They were assured of their privacy and the confidentiality of their information. The consent forms also contained information on how their privacy and confidentiality would be assured. They were given the option of withdrawing from the study if they felt very uncomfortable during the process of data collection. During the planning phase of data collection, the team had a relationship with each of the University Health Centres via the institutions’ administrators. There was a plan to refer any student or staff who may be emotionally unstable as a result of their past sexual violence experience to the school health centre, where they would be managed appropriately. Luckily, we didn’t record any of such events; however, we got a particular questionnaire where a student wrote on it, “Ma, thank you for conducting this study; I had an experience of this with my father about two years ago." Unfortunately, we could not trace this participant because the questionnaire was de-identified.
- The staff working on the project were selected across the six regions of the country. The field supervisors were trained by the research team, and our interaction with them showed that they were excited about the study, and none of them expressed any emotional disturbance resulting from their past victimisation experience. During the training of the field supervisors, efforts were made to inform them of the likely emotional reactions of some participants, who must have been survivors of sexual violence. They were encouraged to refer such participants to the University Health Centre, where they would be managed appropriately. The field supervisors in turn trained the field workers that worked under them.

Whistleblowing context in Nigeria

 Whistleblowing is a term that is just gaining ground in Nigeria. The whistleblowing policy was officially launched by the Federal Ministry of Finance on December 22, 2016, for the purpose of recovering several billions of stolen public funds (Ogbu, 2017). Following the success of the policy in financial systems, it was then recommended in various quarters as a mechanism that could be effective for reporting corruption and other wrongdoings, including sexual violence, in the country. Reporting sexual violence in the Nigerian context is always ‘in the public interest’, especially among young people. We are quite aware that whistleblowing is a legal issue. Whistleblower protection bill was passed in Nigeria on December 14, 2022 (Angbulu, 2022; Dec 14). As of today, in Nigeria, the understanding of whistleblowing is that anyone who is aware of the incident can blow the whistle. However, at the writing stage of the research proposal, we were not aware of any higher education institutions that had domesticated the whistleblowing policy. This particular study was then designed to explore the intention of students and staff in HEIs to use whistleblowing strategies for sexual violence if finally deployed in educational institutions. Looking through the manuscript, we shall ensure that the synonymous use of the terms ‘blow the whistle', 'whistleblower', and' reporting’ is corrected appropriately.

Theoretical Framework

  

- Drawing from the porous nature of the security systems in Nigeria, the stigmatisation of sexual violence survivors, and the victim-blaming attitude in some quarters, it is assumed that whoever wants to blow the whistle for sexual violence must count the cost. Hence, the assertion by the authors that having a positive attitude towards the effectiveness of whistleblowing mechanisms could be a basis for intention for whistleblowing.
- Whistleblowing has been referred to as a planned behaviour, and the Theory of Planned Behaviour has been used by several authors, such as Tarjo et al. (2019), Zakaria et al. (2016), Owusu et al. (2020), and Wahyuni et al. (2021), to support whistleblowing intention. Since our study looked at intention for whistleblowing for sexual violence and also assessed the subjective norm and self-efficacy of the participants, which are precursors to planned behaviour, we are of the opinion that the Theory of Planned Behaviour is the best framework for the study. 

Methods

*Qualitative methods*

- The mixed-gender focus group discussion was adopted because, in class settings in higher education institutions, sexual violence is usually discussed among the students irrespective of their gender. We did not record any issues with the mixed-gender group. The homogeneity of the group is based on participants being officers of students’ and staff’s associations. It is a general observation that males take on more leadership roles than females, which explains why there were more males in the group than females.
- The questions in the FGD guide are not personal to the participants but, general questions. Hence, we are of the opinion that personal interview is not relevant in this case.

*Questionnaire*

 We adapted the questionnaire as stated in the data note: “The questions on attitude, subjective norm, and self-efficacy were adapted from the work of Zakaria et al. (2016). The sexual violence experiences component was adapted from the Sexual Experiences Short Form Victimization (SES-SFV) scale developed by Koss et al. (2006). The SES-SFV scale assessed sexual violence victimisation in the past 12 months and the number of occurrences. The whistleblowing intention component of the questionnaire was formulated using three vignettes, which were adapted from the work of Ahmad (2011).

 The response to ‘Main Concern 1’ above also explains the use of the SES-SFV.

*Vignette*

- The vignette section of the questionnaire depicted the participants as third-party whistleblowers and not as survivors. It is assumed that survivors will report when they are victimised, but a third-party report is not the usual practice in HEIs. Whistleblowing action can be taken by a third party and not necessarily the survivor, and we are of the opinion that the HEIs should be getting used to third party whistleblowing and also take action when there is evidence.
- The rating for the vignette will be explained in the next revision of the manuscript.

*Ethics*

- The class representatives collecting filled-out questionnaires from participants was not a study option but an individual option. The questionnaires were administered to the students in their classrooms, but some insisted they could not fill and submit them immediately; hence, they requested that they would submit to their class representatives for onward submission to the field workers. All efforts to dissuade them from this proved abortive, and we needed to respect their decisions. However, our observation on the field is that some of the participants did not see their responses as so confidential that their colleagues could not have access to them, more so that the bulk of the questions were on whistleblowing intention.
- Other ethical issues are addressed under ‘Main Concern 2’ above.

 
*Pilot Study*

 During the pilot study, the officers of the academic staff association did not participate in the FGD despite various efforts put in place as documented in the published reports (Ogunfowokan et al., 2023). What we did then was to recruit academic staff members who were not necessarily officers to participate in the study. After the pilot study, we planned to use this method in any of the schools in case we ran into similar problems. Fortunately, we never experienced such problems except for a particular school where the entire officers of the association, both academic and non-academic, did not participate hence, we did not conduct FGD in the school.

*Member Checking*

 While the discussion was ongoing, the facilitator reflected on the statements given by the members to confirm their true position. After the discussion, members were asked to exercise some patience while they listened to the selected part of the interview as deemed fit by the facilitator. In fact, in a particular school, the staff group agreed and insisted that a participant must be allowed to record the discussion before the discussion can commence so that if peradventure anything went wrong, they would have evidence of what the discussion was all about. 

Reflexivity statement

- The essence of giving information about the researchers was to ensure that the participants were rest assured that the researchers were university staff and not policymakers or groups of people who may use their responses to indict them.
- The claim ‘to avoid bias’ will be removed from this section.

 
Language use

 Observed inaccurate languages will be corrected in the manuscript.

**References**

 Ado, D. G., Anthonia, E. N. & Babagana, B. (2010). Prevalence and Nature of Sexual Assault among Female Students in a Tertiary Institution in Maiduguri, Nigeria – A Cross Sectional Study. International Journal of Health Research, 3(4), 199-203.

 Adogu, P. O., Adinma, E. D., Onyiaorah, V. I., & Ubajaka, C. F. (2014). Perception, Prevalence and Predictors of Rape among Female Students in a Tertiary Institution South East Nigeria. International Journal of Clinical Medicine, 5(14), 819.

 Akinbode, G. A. & Ayodeji, F. (2018). Sexual Harassment: Experiences, Prevalence and Psychopathology in some Selected Higher Institutions in Lagos, South-West Nigeria. African Journal of the Psychological Studies of Social Issues, 21 (3).

 Angbulu, S. (2022, Dec. 14). FG approves new whistle-blower bill. Punch. https://punchng.com/fg-approves-new-whistle-blower-bill/

 Duru, C. B, Aguocha, C. M.,  Iwu, A. C., Oluoha, U. R.,  Okedo-Alex, I. N., Ikechi, O., & Nwaigbo, E. (2018). Sexual Abuse among Female Undergraduates in Tertiary Institutions in Imo State, Southeast Nigeria: Prevalence, Pattern and Determinants. Open Access library Journal 5(4), ISSN Print:2333-9705; ISSN Online: 2333-9721. Retrieved from: http://m.scirp.org/papers/83742

 Ogbu, S. U. (2017). Whistle Blowing Policy as a Mechanism for Energizing The ‘War Against Corruption’ In Nigeria. International Journal of International Relations, Media and Mass Communication Studies Vol.3 (4) pp.16-32. ISSN 2059-1845(Online); ISSN 2059-1853(Print)

 Onoyase, A (2019). Prevalence of Sexual Harassment of Female Students of Tertiary Education in Taraba State, North East Nigeria: Implications for Counseling. International Journal of Higher Education. 8(1): 77–83. Publisher Full Text

 Tarjo, T., Suwito A., Aprillia, I. D. and Ramadan, G. R. (2019). Theory of planned behavior and whistleblowing intention. Jurnal Keuangan dan Perbankan https://api.semanticscholar.org/CorpusID:150581853

 Zakaria, M., Abd Razak, S. N. A., & Yusoff, M. S. A. (2016). The Theory of Planned Behaviour as a Framework for Whistle-Blowing Intentions. Review of European Studies; 8(3); 2016ISSN 1918-7173 E-ISSN 1918-7181

 Wahyuni, L., Chariri, A., & Yuyetta, E. A. (2021). Whistleblowing Intention: Theory of Planned Behavior Perspectives. The Journal of Asian Finance, Economics and Business, 8(1), 335–341. https://doi.org/10.13106/JAFEB.2021.VOL8.NO1.335

 Owusu, G. M. Y., Bekoe, R. A., Anokye, F. K. and Okoe, F. O. (2020), "Whistleblowing intentions of accounting students: An application of the theory of planned behaviour", Journal of Financial Crime, Vol. 27 No. 2, pp. 477-492. https://doi.org/10.1108/JFC-01-2019-0007

 Ogunfowokan, A. A., Ayotunde, T., Samson-Akpan, P. E., et al.: Intention of Staff and Students to Use Internal Whistleblowing to Report Sexual Violence: A Case Study of a Nigerian University. Journal of Interpersonal Violence. 2023b; 38: 9765–9794. PubMed Abstract | Publisher Full Text
